# Histone Deacetylase Isoforms Differentially Modulate Inflammatory and Autoantibody Responses in a Mouse Model of Myasthenia Gravis

**DOI:** 10.3389/fneur.2021.804113

**Published:** 2022-02-10

**Authors:** Afrin Bahauddin, Maxim Ivannikov, Zhongying Wang, Mohammad Jamaluddin, Kyra Curtis, Naazneen Ibtehaj, Linsey Yeager, Lynn Soong, Xiang Fang, Ruksana Huda

**Affiliations:** ^1^Department of Microbiology and Immunology, University of Texas Medical Branch at Galveston, Galveston, TX, United States; ^2^Optical Microscopy Core, University of Texas Medical Branch at Galveston, Galveston, TX, United States; ^3^Department of Pediatrics, University of Texas Medical Branch at Galveston, Galveston, TX, United States; ^4^School of Medicine, University of Texas Medical Branch at Galveston, Galveston, TX, United States; ^5^Department of Neurology, University of Texas Medical Branch at Galveston, Galveston, TX, United States

**Keywords:** acetylcholine receptor, histone deacetylase, IL-6, myasthenia gravis, autoantibody, autoimmunity

## Abstract

Myasthenia gravis (MG) is an autoimmune disease characterized by chronic muscle fatigue and weakness caused by autoantibodies and complement-mediated damage at neuromuscular junctions. Histone deacetylases (HDACs) are crucial epigenetic regulators of proinflammatory gene expression; however, it is unclear whether HDACs modulate chronic inflammation or autoantibody production associated with MG pathogenesis. We examined expression profiles and serum levels of key inflammatory cytokines (IL-6 and IL-21) and acetylcholine receptor (AChR)-specific autoantibodies following pharmacological inhibition of key HDAC isoforms in a mouse model of MG. We found that HDAC inhibition significantly reduced the production of IL-6, but not IL-21, in AChR-stimulated PBMCs and splenocytes (*n* = 5 per group). Trichostatin (pan-HDAC inhibitor) treatment of MG-PBMCs (*n* = 2) also exhibited reduced production of induced IL-6. Although HDAC1 inhibition lowered IL-6 levels the most, HDAC2 inhibition depleted intracellular IL-6 and markedly reduced serum anti-AChR IgG2b in EAMG mice. The transcriptomic profiling and pathway mapping also revealed that autoimmunity-linked, major cell signaling pathways were differentially altered by HDAC1/2 inhibition. HDAC inhibition-mediated reduction in IL-6 and autoantibody levels also correlated with milder disease and preservation of muscle AChR in the treated mice. Overall, our findings revealed isoform-specific functional variance of HDACs in reducing inflammation and identified HDAC-regulated many genes underlying specific inflammatory and autoantibody pathways in EAMG. Thus, the study provides a rationale for further research to evaluate the HDACs or their gene targets as a potential adjunct treatment for MG.

**Graphical Abstract:**
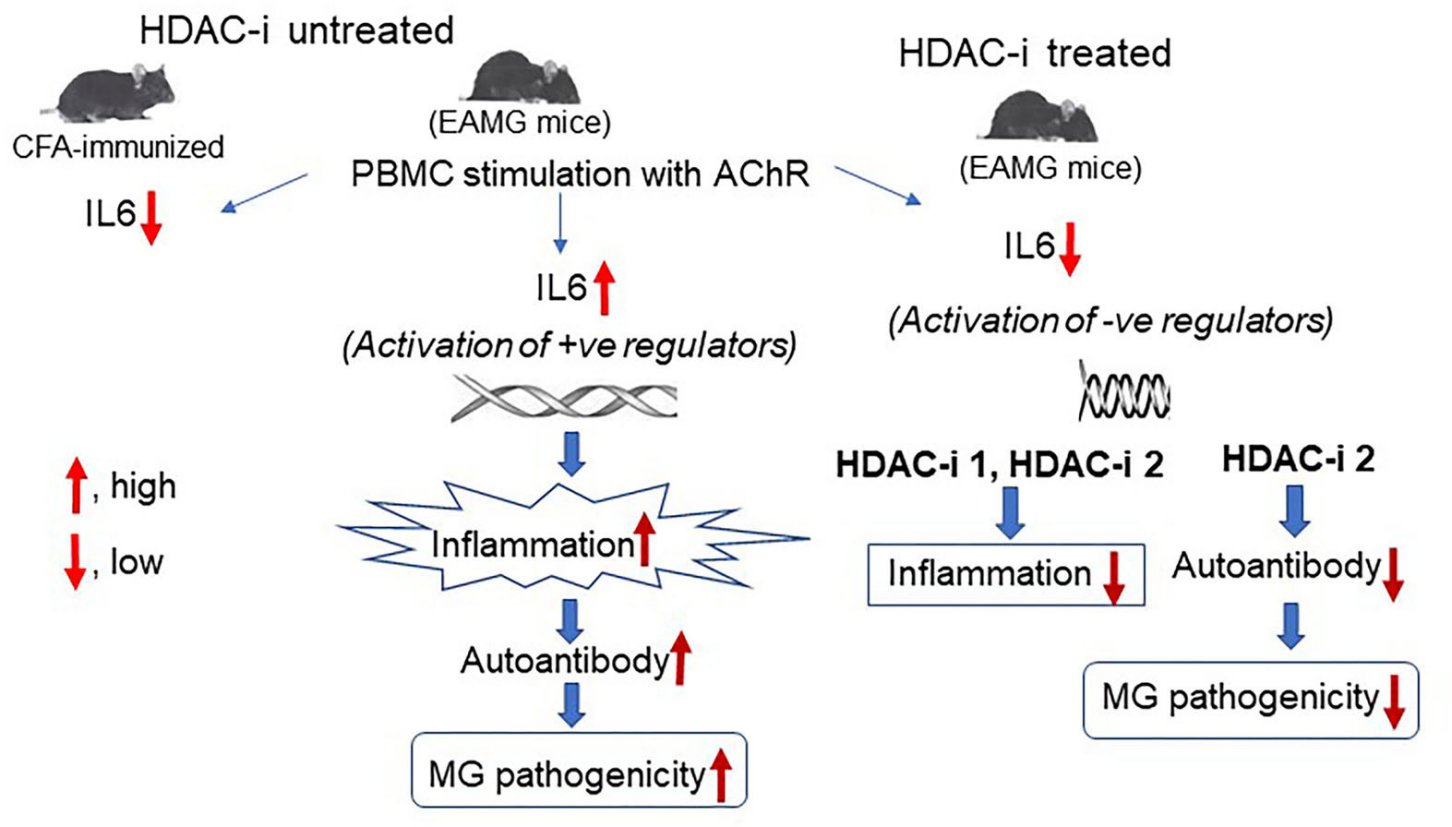
In Brief. Schematic effects of inhibition of HDAC isoforms on production of IL-6 and the anti-AChR autoantibody in MG. Deacetylation-mediated activation of specific histone leads to the activation of genes and signaling pathways that induce IL-6 expression in MG. Conversely, inhibition of histone deacetylation through activation of negative regulators of IL-6 transcription, leads to the repression of IL-6 transcript levels that results in reduced activation of B cells, low levels of autoantibody, and disease improvement.

## Introduction

Myasthenia gravis (MG) is a debilitating autoimmune disease characterized by muscle fatigue, caused by autoantibodies (autoAbs) and complement-mediated damage at the postsynaptic neuromuscular junction (NMJ) (1, 2). Proinflammatory mediators that elicit Th1 and Th2 responses play a prominent role in MG pathogenesis (3). Of these, IL-6 and IL-21 and their downstream activities have been strongly linked to MG development (4, 5). IL-6, a pleiotropic cytokine, can bind to membrane-bound receptor or soluble IL-6 receptors (sIL-6R), which then signals through membrane glycoprotein 130 (GP-130). Subsequent activation of the Janus kinase/signal transducer and activator of transcription (JAK/STAT) pathway by GP130 induces the expression of IL-6-dependent genes (e.g., CCl2), leading to biological responses (6, 7). In general, most interleukins are known to stimulate Ab production *via* priming and activation of dendritic cells, antigen-specific T-helper cells, B cells, and pathogenic differentiation and development of plasma B cells (8). In addition, IL-6 can help differentiate Th17 from Treg cells and prolong plasma cell longevity (9). Increased IL-6 production has been detected in thymic epithelial cells and activated B cells of MG patients (10). Our group and others have also demonstrated that IL-6-deficient mice are resistant to MG, and that anti-IL-6 Ab treatment reduces autoAb levels and suppresses the disease in a rat model of MG (11, 12). Therapeutic Abs against IL-6 or IL-6 receptors have been developed and shown to be beneficial for patients with refractory MG (13), further providing the evidence that IL-6 play a pivotal role in MG. IL-6-mediated upregulation of IL-21 and the role of IL-21 in MG pathogenesis have recently been demonstrated (14, 15). Elevated serum levels of IL-21 secreted from follicular T-helper cells have been shown to correlate with enhanced Ab production from B cells in MG patients (16). IL-21 upregulation has also been reported in stimulated PBMCs from patients positive for either AChR-specific or muscle-specific kinase (MuSK)-specific autoAbs (17).

Increasing evidence suggests a prominent role of histone deacetylase (HDAC) in interleukin production (18, 19). HDACs normally prevent gene transcription by blocking the acetylation of DNA-binding histones. Therefore, HDAC inhibition favors histone acetylation by histone acetyltransferases (HATs). Acetylation of histones on specific lysine residues (e.g., H3K9 and H3K14) allows the access of RNA polymerase II to gene promoter (s), facilitating gene transcription (20). In contrast, H4K20ac has recently been shown to mediate the repression of several genes (21). In addition, a recent study demonstrated that nonhistone proteins (e.g., HMG, p53, and α-tubulin) are also the substrates of many HDACs that mediate their posttranslational modifications and biological activities (22). Intriguingly, HDAC inhibitors (HDAC-i), but not HDAC agonists, have been shown to repress rather than enhance transcription of many genes implicated in disease pathogenesis. A likely explanation is the activation of one or more specific genes by HDAC-i, which in turn represses gene(s) associated with the inflammatory response, or HDAC-i-mediated posttranslational modification and biological activities of proinflammatory or anti-inflammatory nonhistone proteins (23).

Recent evidence suggests that many HDAC-i, sourced from natural products, exhibited substantial therapeutic benefits with minimal to no adverse effects in clinical trials and a few FDA-approved treatments (24). Isoform-specific HDAC-i or pan-HDAC-i ameliorated many autoimmune diseases, including rheumatoid arthritis, colitis, systemic lupus erythematosus, autoimmune encephalomyelitis, multiple sclerosis, and Duchenne muscular dystrophy by suppressing inflammation in patients or in animal models (25–30). However, little is known whether the disease improvements occur, at least partly, through the mitigation of autoAb responses or altered expression of genes sensitized by HDAC inhibition. The inhibition of chronic inflammation and autoAb represents a viable approach also for MG therapy. Therefore, in this study, we examined the effects of HDAC isoform inhibition on the inflammatory and autoAb responses and conducted mRNA profiling to identify autoimmune-associated target genes of HDACs in a mouse model of MG (EAMG).

## Materials and Methods

### Animals

C57Bl6/J male mice (8 weeks, ~20g) obtained from Jackson Laboratory (Bar Harbor, Maine) were housed and maintained in a barrier facility. This animal study was performed as per the National Institute of Health and UTMB Animal Care and Use Committee guidelines.

### EAMG Disease Induction

The mice were immunized 4 weeks apart in the footpad and shoulder with affinity-purified torpedo AChR (20 μg each) in complete Freund adjuvant (CFA) or CFA alone. Following two booster immunizations, CFA/AChR (CA)-immunized mice showed the clinical signs of muscle weakness. EAMG disease was confirmed in mice by blinded observation of the clinical grades or weaker grip strength of the forelimbs and increased titers of the autoAb in CA-immunized mice (31).

### HDAC-i Treatment of EAMG Mice

MG disease in mice was clinically graded as follows: grade 0, normal muscle strength; grade 1, weak at exercise and hunched posture with reduced mobility; grade 2, weakness at rest; grade 3, moribund and paralyzed. Two weeks after second booster immunization, EAMG mice with grade 2/3 disease (31) were randomly assigned into groups (five per group) for treatment. To examine whether HDAC isoforms are involved in the regulation of IL-6 or IL-21, EAMG mice were administered intraperitoneally with the following isoform-specific HDAC-i at a dose of 10 mg/kg body weight: *MS-275 for HDAC1 and 3, CL-994 for HDAC1, MC1568 for HDAC2*, and *BRD9757* (cross-reactive to mouse) *for HDAC6/8* (Sigma Aldrich, MO). DMSO (solvent at <0.1%) or PBS were used as controls. Dosages of HDAC-i were selected as per manufacturer's recommendations based on previous studies cited in their brochure or PubMed. The mice were treated thrice, 10 days apart for prolonged exposure of mice to HDAC-i for any anti-inflammatory effects, and later to examine the associated clinical outcomes. Tail-vein blood was collected from the mice at time points: 24 h post 1st, 72 h post 2nd, 72 h and 4 weeks post 3rd treatments. The treatment outcomes on the disease grades were recorded thrice a week, post treatment. The mice were euthanized 4 weeks post 3rd treatment to collect the spleen, forelimb muscle, lymph nodes, and carcasses. The cells and tissues used were either fresh or fixed in 4% formaldehyde or stored at −80°C.

### Cell Isolation, PBMC/Splenocytes Stimulation, and ELISA

PBMCs or splenocytes (>4 × 10^6^ cells) were processed using Lymphoprep on SepMate tubes (StemCell Technologies, MA). Cells were treated with the RBC lysis solution (Sigma) to remove contaminating red blood cells and washed with PBS containing 2% heat-inactivated FBS.

To enhance cytokine expression, PBMCs were stimulated with either AChR (MG-specific antigen, affinity-purified from torpedo electroplax organ) at 1 μg per 1 × 10^6^ cells or a nonspecific stimulant, PMA/ionomycin (Sigma) at 10 ng/ml of medium. Cells were resuspended in RPMI supplemented with 2% heat-inactivated FBS and incubated with AChR, PMA/ionomycin, or PBS for 16 h or 26 h at 37°C in an incubator with 5% CO_2_ and 95% humidity. The relative levels of induced IL-6 and IL-21, secreted in the culture supernatant, were measured by ELISA (IL-6 Quantikine ELISA kits, R and D Systems, MN; MO IL-21 COATED ELISA, ThermoFisher Scientific, MA) using slight modification of sample dilutions. Cells were lysed to isolate RNA, which was further processed for quantitative RT-PCR to detect the relative abundance of cytokine mRNA. Aliquots of cells were also used for flow cytometry analyses.

### HDAC Inhibition in Human PBMCs

For a feasibility test, PBMCs were collected from clinically diagnosed patients with MG (*n* = 2) seropositive for 127 anti-AChR IgG, and not in remission. Patient one was a 54-year-old male with positive ACh binding and blocking, modulating antibody but no Anti MuSK Ab. He had spontaneous diplopia with MG severity score of three (https://myasthenia.org/). He could not tolerate pyridostigmine, prednisone and was treated with IVIG and plasma exchange one time for exacerbation before. He did not receive any immunosuppressive medication at the time of blood collection. Patient two was a 62-year-old male with ACh binding and blocking, modulating antibody (ARUP Laboratories, Salt Lake City, UT). He had newly diagnosed MG with mild diplopia, moderate ptosis, mild facial weakness, minimal coughing, and mild dysphagia with MG severity score of 5. His Anti-MuSK was not tested and was on pyridostigmine. The sample was collected at the time of the first clinical visit and before any treatment interventions.

The control group (n = 2) comprised age- and gender matched healthy donors (53- and 62-year-old males) with no known diseases. This pilot study was approved by the institutional review board of the UTMB, and informed written consents were obtained from the patients.

Purified PBMCs from patients with MG and healthy donors were treated with Erythrocyte Lysing solution (R&D Systems), washed in PBS and 2% FBS (ATCC, VA), and resuspended in RPMI 1,640 supplemented with glutamine (ATCC) and 10% FBS. Cells (4 × 10^6^ per well) were incubated at 37°C with 5% CO_2_ and treated with 50 ng/ml and 100 ng/ml medium using a 2 μg/μl stock solution of pan HDAC-i, trichostatin A (TSA), or DMSO for 24 h. The supernatant was removed, and the cells were resuspended in RPMI with 2% heat-inactivated FBS. TSA treatment was followed by stimulating cells with torpedo AChR (98% sequence homology with human AChR) 1 μg per 1 × 10^6^ cells for 24 h. The supernatant was collected to measure the relative levels of IL-6 using Human IL-6 Quantikine ELISA kit (R&D Systems) following pan HDAC inhibition.

### Gene Expression, Enzymatic Activities, and Histone Acetylation Assays

RNA and cDNA were prepared from PBMCs and the spleen using TRIzol and Superscript III (kits, Invitrogen, MA). Quantitative RT-PCR was performed using Fast-Advanced TaqMan Master mix and IL-6- and HDAC- specific Fam-labeled probe-primers/TaqMan Gene Expression Assays (Thermo Fisher Scientific, MA) on a Bio-Rad CFX 96 system. Relative fold changes in HDACs and IL-6 mRNAs normalized against beta-actin mRNA (standard) were computed using the CFX software (2^−Δ*ΔCt*^ method) (Bio-Rad). In addition, the altered HDAC activity in the PBMC lysates of EAMG mice (treated or not with HDAC-i) was confirmed using HDAC activity assay kits (Active Motif, Inc., CA). HAT activities in the samples were also measured using a HAT assay kit (Active Motif). The fluorescent intensities of the samples were measured by using FLUOstar OPTIMA (BMG LABTECH Inc., NC) at an excitation 355 nm and emission 460 nm wavelengths.

Whole-cell lysates of splenocytes were sonicated and centrifuged at 4°C. Following quantitation and the addition of 4X loading buffer, the protein was resolved in 4–20% SDS-PAGE and immunoblotted using H3K4ac (Abcam, MA) and H4K20ac (RevMAb Biosciences, CA) specific primary Abs (Abcam) at 1:100 and 1:1000 dilutions respectively, and then mouse antirabbit secondary IgG-HRP Abs (1:5000; Amersham, NJ). ECL (Pierce, IL) was performed. The gel image was captured using GE Amersham Imager 680 and each band density was measured using VisionWorks software on a UVP Imaging system (Analytik Jena US LLC, CA). The data represents the results of three replicate experiments.

### Flow Cytometry Analysis

PBMCs and splenocytes were prepared for flow analysis as per our published protocol (32). Briefly, cells were isolated as single-cell suspensions, stimulated with AChR, or PBS in presence of brefeldin (Sigma) 0.2 μM (to partially block cytokine transport), washed with PBS containing 2% FCS, Fc-blocked, prefixed and permeabilized for intracellular cytokine, and either surface-stained or stained with FITC-, PE-, PE-cy7, eFluor 450, APC, and other fluorochromes-labeled Abs. Cells were either analyzed immediately or fixed in 2–4% paraformaldehyde. All cells were analyzed for the intracellular expression of IL-6 in B cells, T cells, dendritic cells, and monocytes. Plasma cell frequencies were determined in an aliquot of splenocytes (not stimulated with AChR) stained with CD269 (B cell maturation antigen, BCMA)- specific monoclonal Abs (Ray Biotech, GA) and PE-labeled secondary Abs (eBioscience, CA). All cells were analyzed on a BD LSR Fortessa Flow cytometer using FACSDiva software (BD Bioscience, CA) in the UTMB Flow Cytometry and Cell Sorting Core.

### Anti-AChR Ab Measurement

Serum levels of anti-AChR IgG2b (predominant and pathogenic isotype) in EAMG mice were measured using ELISA 72 h post 3rd treatment using our affinity-purified mouse muscle AChR (coating antigen), rat antimouse IgG2b-HRP (secondary Ab; BD Bioscience, CA), and ABTS (2'azino-bis 3-ethylbenzthiazoline-6-sulfonic acid) (Invitrogen, MA) as substrate (33). To check if HDAC inhibition***-***mediated Ab reduction had a time-dependent reversal, EAMG mice were bled at 4 weeks post 3rd (last) treatment. Serum was used at a pre-titrated dilution of 1:1000.

### Nonradioactive Quantitation of Muscle AChR

Functional muscle-AChR content reliably represents the treatment effect on muscle strength of EAMG mice (33). We standardized a new ELISA method alternative to radioimmunoassay for quantitating the relative abundance of functional AChR in the proximal forelimb muscles that are affected most in EAMG. First, EAMG serum-coated plates were blocked with 2% BSA and incubated at 37°C for 1.5 h with an equal amount (total protein) of supernatant from the muscle-membrane extracts (*n* = 3 for CFA, *n* = 5 for each PBS, DMSO, and HDAC-i treated EAMG group). Next, the plate was washed and incubated again with biotinylated α-Bungarotoxin (Thermo Fisher Scientific) for 1 h. Subsequently, streptavidin-HRP (Thermo Fisher Scientific) was added to read the plate at 405 nm using ABTS as substrate.

The result was further verified by a second method, using Alexa-555-conjugated bungarotoxin and measuring the fluorescence at an emission 565 nm using a fluorescence plate reader, Cytation 5 (BioTek, VT) (data not shown).

### mRNA Profiling

To analyze genes sensitive to HDAC inhibition, total RNA was extracted from a portion of snap-frozen spleen using Qiagen RNeasy Mini kit (Qiagen, MD). For nCounter Autoimmune profiling of genes (NanoString Technologies, WA), the manufacturer's protocol (performed by the provider) included the following steps: hybridization of RNA to the reporter and capture probes in a thermocycler, removal of excess probes, purification, and immobilization of processed RNA onto a sample cartridge, and documentation of normalized relative fluorescent signals (specific mRNA copies) on a nCounter Digital analyzer. We analyzed the data using nSolver 4.0 software per manufacturer's instructions.

### Immunofluorescence Imaging

Paraffin-embedded spleen sections (CA, Hi-1, Hi-2) were treated with xylene, fixed in ethanol, washed, blocked with 5% goat serum or 2% BSA, and incubated at 4°C overnight with mouse-specific primary Abs for CDK9, IAP-1 and ISG-20. The slides were washed and further incubated for 2 h with secondary Abs conjugated to Alexa 555-, Alexa 350- and Alexa 488-conjugated anti-mouse IgG isotypes (1:100) (Invitrogen). After washing in PBS, the sections were air-dried and photographed with an Olympus IX-70 microscope (10X, 20X) equipped with a DP-11 digital camera (33). Fluorescence deposits from 10 microscopic fields of the tissue sections from 3 samples per group were counted. Mean numbers from each group were plotted for relative quantitation of the proteins.

### Statistics

Each experiment was repeated 3 times. All data were compared and evaluated using one-way ANOVA followed by Holm-Sidak or Turkey's *post hoc* analysis where applicable. Calculated *P*-values were considered *P* < 0.05 as significant. The Biostatistics Core at UTMB further performed power analyses for each experiment. All statistical testing assumed a 95% level of confidence.

## Results

### sIL-6 and sIL-21 Levels From AChR-Stimulated PBMCs

**Figure 1:**
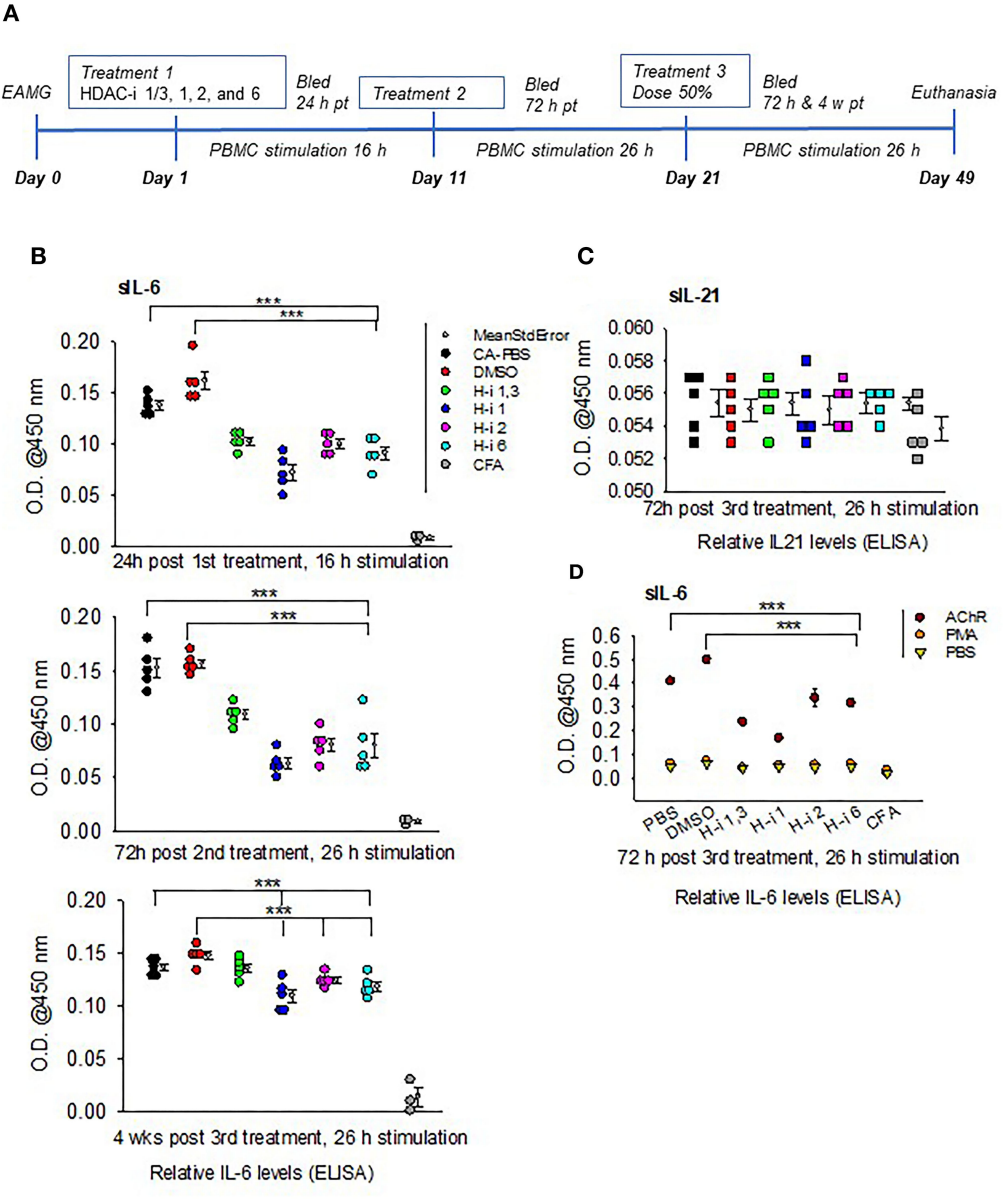
Relative levels of secreted IL-6 and IL-21 in the supernatants of AChR-stimulated PBMCs. **(A)** Treatment schematic of EAMG model (grade 2) with HDAC inhibitors (HDAC-i). **(B)** Relative IL-6 levels in the supernatant upon stimulation of PBMCs with AChR at the indicated post treatment time points. **(C)** Relative IL-21 levels in the supernatant of AChR-stimulated (26 h) PBMCs from EAMG mice treated or not with HDAC-i. **(D)** Relative levels of IL-6 secreted from PBMCs stimulated for 26 h with specific (AChR) or nonspecific stimulus (PMA/ionomycin) or no stimulus (PBS-only). PBMCs were isolated 72 h later from untreated EAMG mice or EAMG mice treated with HDAC-i (post 3rd treatment). Each dot represents mean +/- standard deviation; readings are from triplicate microplate wells belonging to the same group of pooled supernatant samples. CFA (*n* = 3) and each EAMG (*n* = 5 per group). ***P < 0.001, ANOVA. The experiment is representative of three similar experiments.

A marked reduction in the levels of secretory IL6 (sIL-6) was detected following 16 h stimulation of PBMCs obtained from mice treated for 24 h with HDAC-i. The reduction was significant for all groups treated with HDAC-i compared to groups treated with DMSO or PBS (Figures 1A,B). Comparable levels of induced sIL-6 were present in the supernatant of PBMC samples obtained at 72 h post 2nd HDAC-i treatment and stimulated with AChR for 26 h (increased duration was used to enhance cytokine production). To monitor the persistence of responses to HDAC-i and the potential effect of dose reduction, EAMG mice were then treated with 50% doses of HDAC-i on their 3rd treatment, and PBMCs were collected at two time points, 72 h and 4 weeks post this treatment. Without further treatment, the suppressive effect of HDAC-i on inducible sIL-6 was considerably diminished 4 weeks post 3rd treatment (Figure 1B). Inducible sIL-6 from AChR-stimulated PBMCs was still significantly decreased in the HDAC-i (1, 2, and 6) treated EAMG groups than the PBS and DMSO-treated control groups. Unlike sIL-6, secretory levels of IL-21 in the supernatant were barely detectable and remained unaltered among the groups at 72 h post 3rd treatment (Figure 1C).

To further confirm that the sIL-6 induction in the PBMCs is antigen (AChR)-specific, PBMCs isolated 72 h post 3rd treatment from EAMG mice were stimulated with AChR, PBS, or a nonspecific stimulant, PMA/ionomycin for 26 h. There were nearly undetectable levels of sIL-6 in the supernatant of PBMCs stimulated with PBS or PMA/ionomycin compared to that in the supernatant of PBMCs stimulated with AChR (Figure 1D).

### IL-6 mRNA Expressions in AChR-Stimulated PBMCs

**Figure 2:**
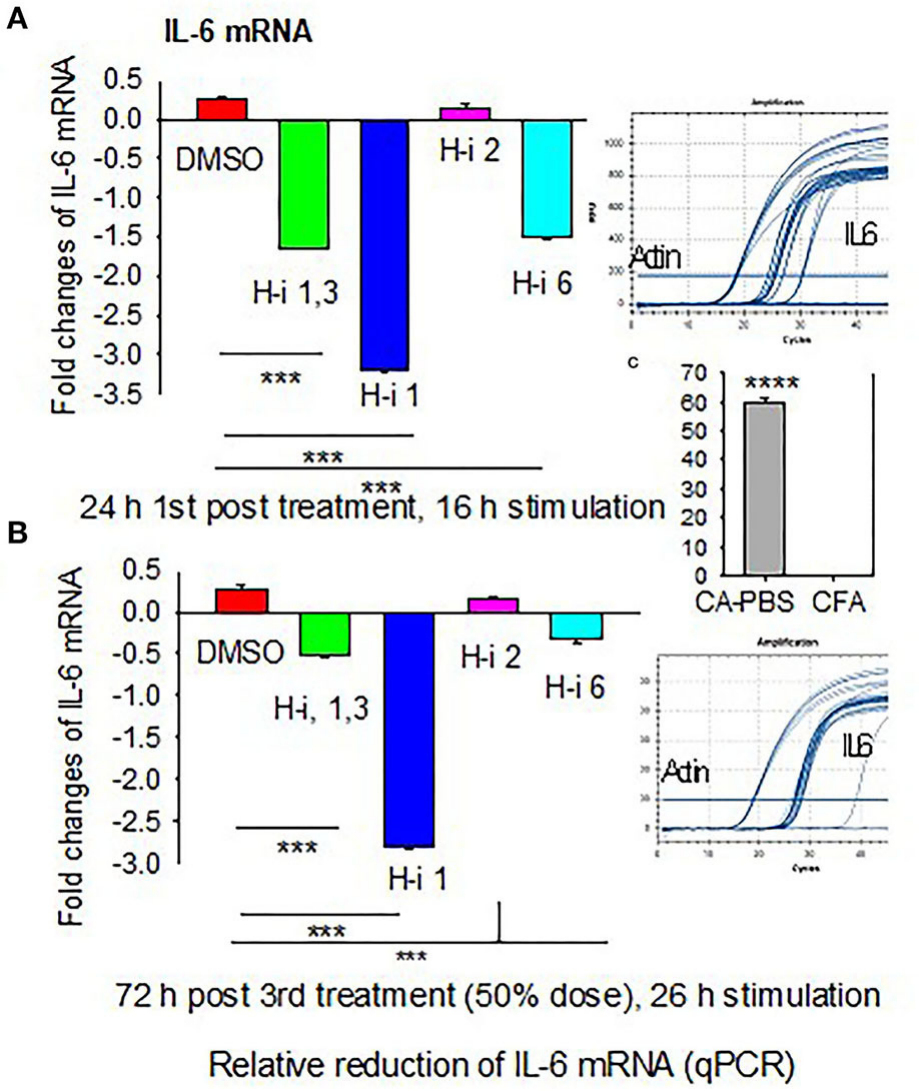
Fold changes in IL-6 mRNA in AChR-stimulated PBMCs. PBMCs were isolated from CFA or CFA/AChR (CA)-immunized EAMG mice treated or not with HDAC-i and stimulated with AChR. **(A)** Bar graph depicts relative fold changes (low on the bottom and high on top part in the plot) of IL-6 mRNA in stimulated PBMCs over CA 16 h post 1st treatment. **(B)** Fold-relative IL-6 mRNA 72 h post 3rd treatment. Sample amplification plots in rectangles on the right side of plots A and B. **(C)** Representative fold-relative IL-6 mRNA in stimulated PBMCs collected from PBS-treated CA (EAMG) vs. CFA mice (60 to 1000-fold higher in EAMG over CFA) (*n* = 5 per group), ***P < 0.001, ANOVA. The experiment is representative of three similar experiments.

To determine whether AChR-induced sIL-6 levels correspond to the IL-6 mRNA levels, RNA was isolated from AChR-stimulated PBMCs at 24 h post 1st treatment and again at 72 h post 3rd treatment. Quantitative RT-PCR revealed that IL-6 expression corresponded to sIL-6 levels (Figures 2A,B). Fold reduction in IL-6 mRNAs was higher in response to HDAC-i 1 treatment compared to other HDAC-i treatments. The slight increase in IL-6 levels post 1st treatment with HDAC-i 2 was not significant. Decreased IL-6 mRNA levels also corresponded to a 50% dose reduction in samples from the 3rd treatment. Notably, AChR stimulation of PBMCs isolated from PBS-treated EAMG mice induced robust IL-6 mRNA expression (over 60-fold higher) than that seen in similarly induced PBMCs from CFA-immunized mice (Figure 2C).

### Expression and Enzymatic Activities of HDAC Isoforms in CFA vs. EAMG Mice

**Figure 3:**
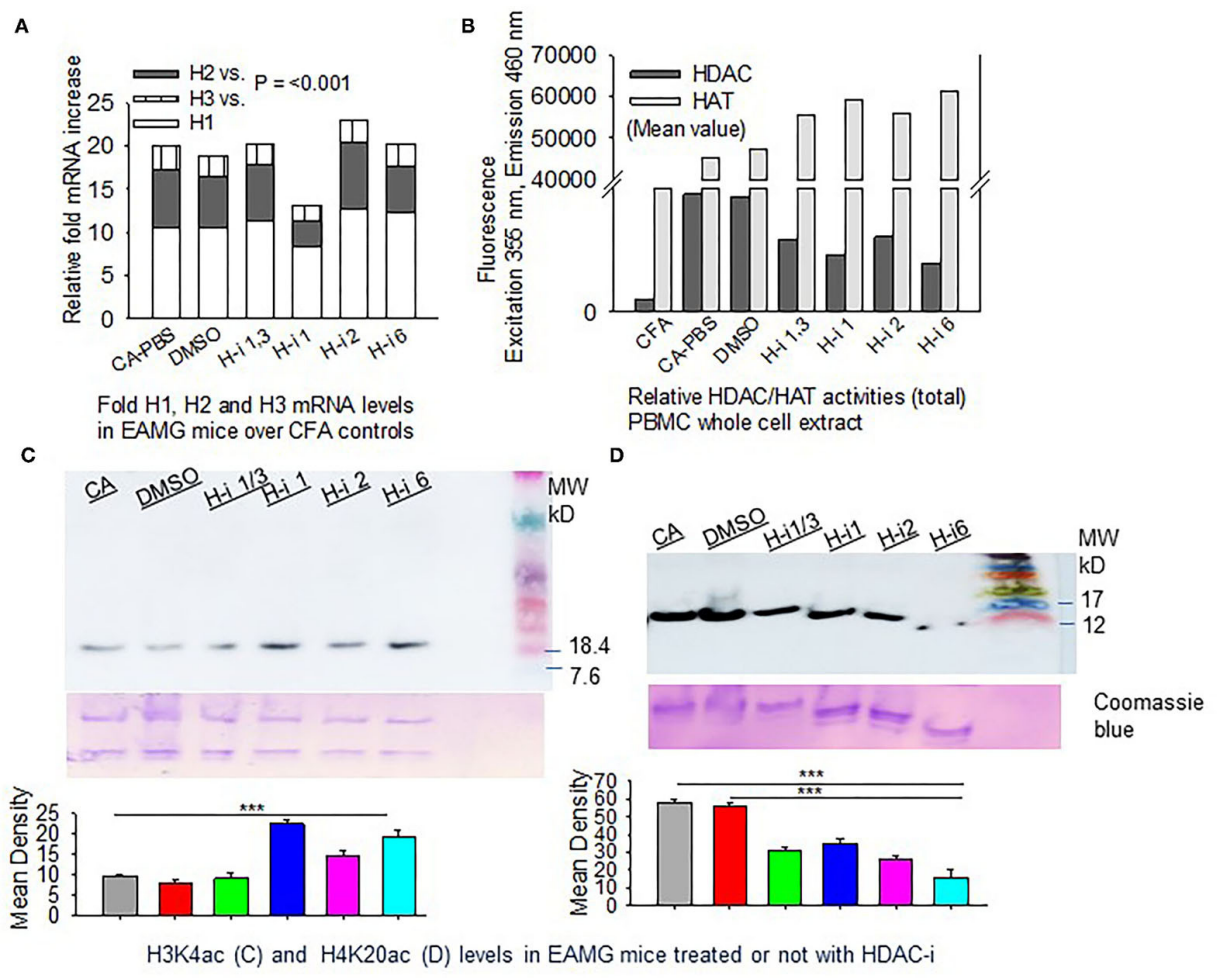
HDAC expression and HDAC vs. HAT activities in PBMCs. CFA and EAMG mice (± HDAC-i) were bled 72 h post 2nd and 3rd treatments. **(A)** Relative mRNA fold changes of HDAC isoforms 1, 2, and 3 in AChR-stimulated PBMCs from post 3rd treatment. **(B)** Relative HDAC/HAT total enzymatic activities in the whole-cell extract of AChR-stimulated PBMCs from post 2nd treatment. Data (fluorescence intensity) shown are the mean values of the pooled samples in wells assayed in duplicate (*n* = 3 for CFA; *n* = 5 for CA-PBS and each treatment groups). **(C,D)** Immunoblots showing H3K4ac and H4K20ac in whole-cell lysates of splenocytes from EAMG mice treated or not with HDAC-i. Bottom panel: nonspecific bands to show loading uniformity in the gel stained with Coomassie blue. The experiment was repeated thrice. ***P < 0.001, ANOVA.

We then investigated the expression levels of HDACs (1, 2, and 3) and their total enzymatic activities in EAMG mice. The CFA-AChR (CA) immunized mice, with or without HDAC-i treatment, expressed approximately 10.99 ± 0.63, 5.87 ± 0.66, and 2.33 ± 0.14-fold higher baseline mRNA levels of HDAC1, HDAC2, and HDAC3, respectively, than the CFA-immunized mice (Figure 3A). As the measurement of enzymatic activity is considered more reliable method to assess HDAC inhibition, we next quantified the changes in the total HDAC activity after HDAC inhibition. A robust increase in HDAC activity was observed in the PBMC lysates of PBS-treated CA-immunized mice compared with those of PBS-treated CFA-immunized mice. Treatment of the groups with HDAC-i significantly reduced total HDAC activities with a corresponding rise in HAT activities, as indicated by altered fluorescence in the PBMC lysates obtained from the respective groups (Figure 3B). Interestingly, the inhibition of HDAC isoforms increased the acetylation of H3K4 but decreased it at H4K20 residues in splenocytes (Figures 3C,D).

### HDAC Inhibition in Human PBMCs

**Figure 4:**
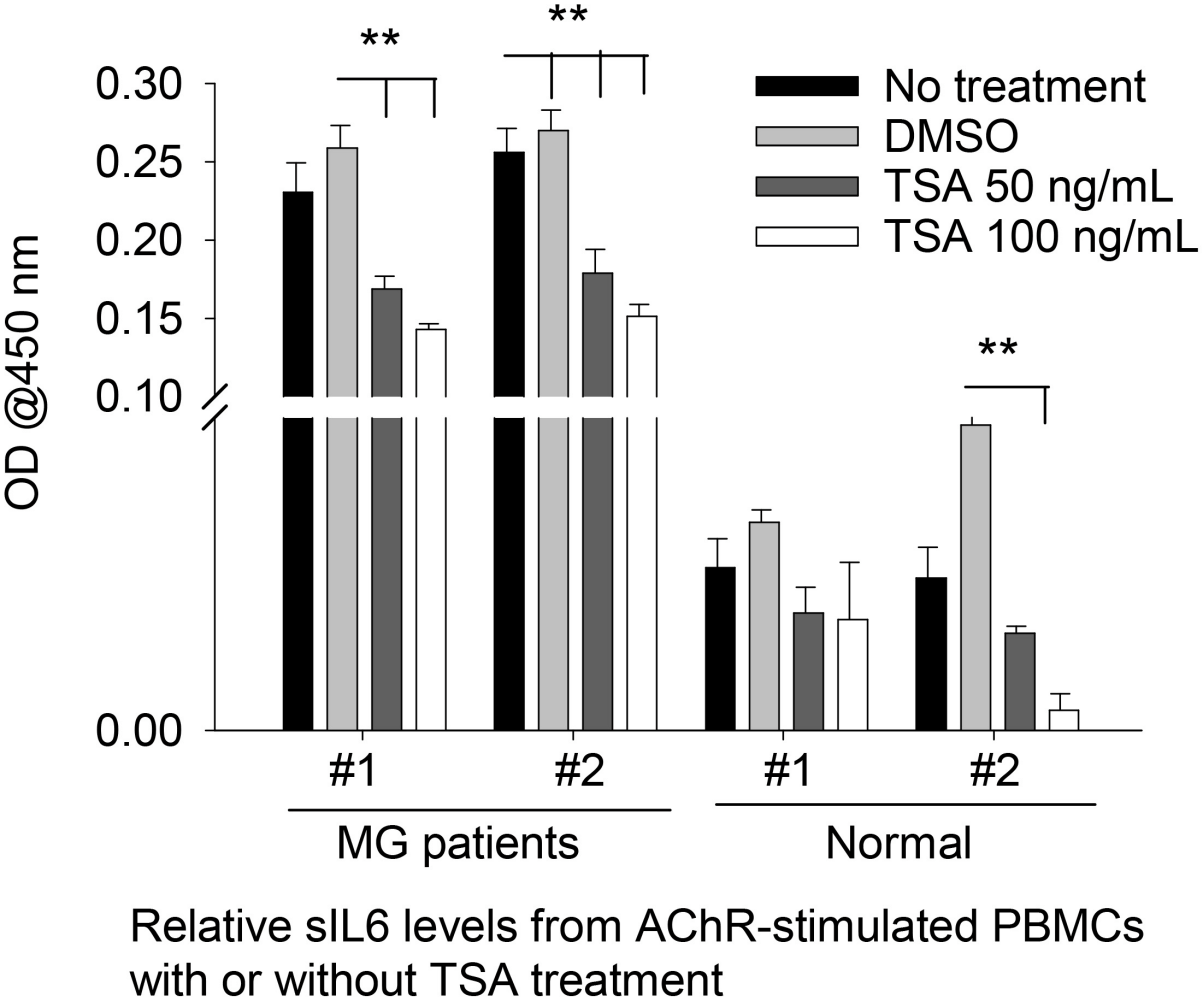
Secreted IL-6 levels from human PBMCs treated with pan HDAC-i. Purified PBMCs from MG patients and healthy donors (*n* = 2 per group) were incubated with TSA at 100 ng/ml and 50 ng/ml medium *ex vivo* for 24 h and then stimulated with AChR for 16 h. Secreted levels of IL-6 were then measured *via* ELISA. **P < 0.01, ANOVA. The experiment was performed once.

As *ex vivo* response of human PBMCs to different HDAC-i cannot be compared to the *in vivo* response of EAMG mice or MG patients (*n* = 2) to the HDAC-I treatments, we chose to treat MG-PBMCs with Trichostatin (TSA), which is a pan-HDAC inhibitor for HDAC 1, 2, 3, and 6. Purified PBMCs (4 × 10^6^) from patients with MG and healthy donors were incubated with or without TSA or DMSO for 24 h and then stimulated with AChR for 16 h. The relative levels of IL-6 in the supernatant of PBMCs, as determined by ELISA, showed similar trends as that in the supernatant of EAMG-PBMCs treated with HDAC-i (Figure 4). The sIL-6 levels in the supernatant were markedly decreased for MG-PBMCs pretreated with both doses of TSA *ex vivo*. AChR stimulation of MG-PBMCs showed significantly elevated level of sIL-6, whereas induced PBMCs isolated from the healthy donors did not significantly increase IL-6 and were detected near baseline levels (PBS- or DMSO-treated).

### Cell-Specific IL-6 Repression by HDAC-i Treatment

**Figure 5:**
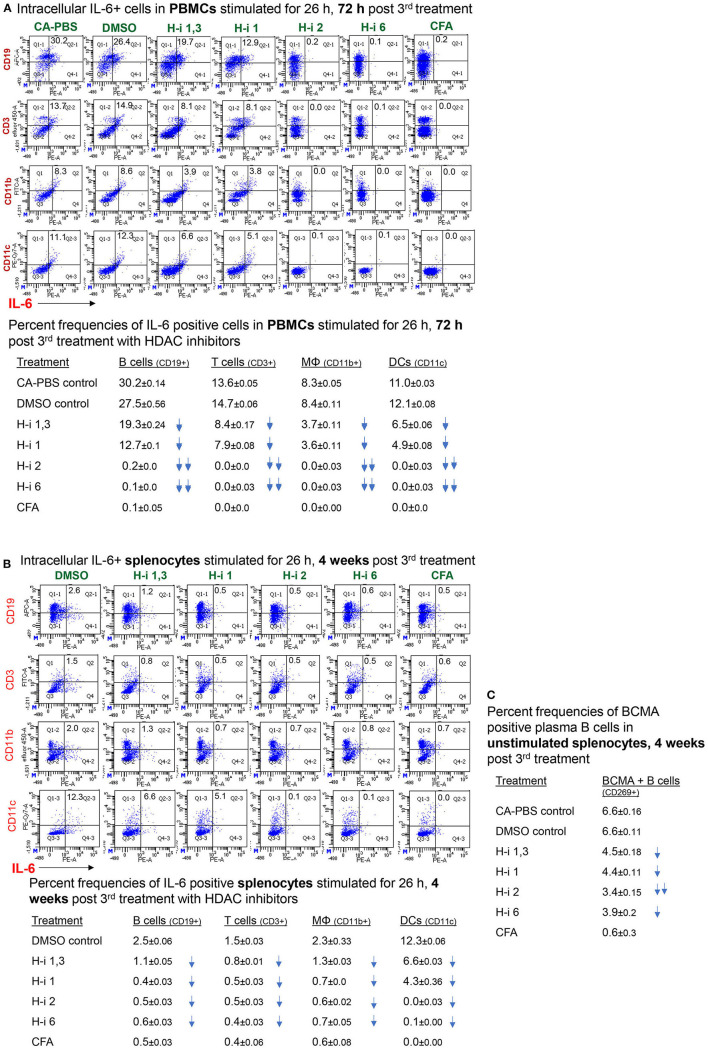
Frequency of (intracellular) IL-6-positive immune cell types. **(A,B)** CFA (*n* = 3) or EAMG mice (*n* = 5 per group) treated with HDAC-i were bled 72 h and 4 weeks post 3rd treatment for flow cytometric analyses of IL-6-producing cells. Isolated PBMCs and splenocytes were stimulated with AChR in presence of brefeldin for 26 h, fixed/permeabilized, and stained with specific fluorochrome-conjugated antibodies to enumerate IL-6-positive immune cell types. **(C)** An aliquot of splenocytes from EAMG mice (isolated 4 weeks post 3rd treatment) treated with HDAC-i and not stimulated but Fc-blocked and stained with BCMA-specific monoclonal Ab (Ray Biotech) and PE-conjugated secondary Ab. The numbers below the figures summarize the percent frequency changes of immune cells. Shown on the right are the percent frequencies of BCMA-expressing cells. The experiment is representative of three similar experiments. Three replicates of pooled samples per group were used for flow analyses.

To assess cell-specific IL-6 repression by HDAC inhibition in EAMG mice, we enumerated the frequency of immune cells positive for intracellular IL-6. Flow cytometry analyses of stimulated PBMCs and splenocytes revealed IL-6+ populations in all immune cell types. Notably, the number of intracellular IL-6-expressing B cells (CD19^+^), T cells (CD3^+^), macrophages (CD11b^+^), and dendritic cells (CD11c^+^) in PBMCs were virtually nonexistent in EAMG mice treated with HDAC-i 2 and HDAC-i 6 (Figure 5A). The effect of HDAC-i started fading after the discontinuation of treatment following the 3rd treatment. We next assessed the IL-6^+^ cell frequencies in splenocytes of EAMG mice. There were no significant numerical differences in intracellular IL-6^+^ splenocytes among the different treatment groups, except for HDAC-i 1/3 treated group that still presented 40% or more IL-6^+^ cells (excluding macrophages) 4 weeks post 3rd treatment. Each treatment group, however, exhibited a significantly lower number of IL-6^+^ immune cell types (Figure 5B) compared to PBS- or DMSO-treated EAMG controls. To examine the effect of HDAC-i, specifically HDAC-i 2, on the number of plasma cells that exclusively express B-cell maturation antigen (BCMA) receptors, we enumerated BCMA^+^ B cells in unstimulated splenocytes. BCMA^+^ cell frequencies also markedly declined in the HDAC-i-treated groups, particularly in the group treated with HDAC-i 2 (Figure 5B).

### Effect of HDAC Inhibition on AChR-Specific autoAb

**Figure 6:**
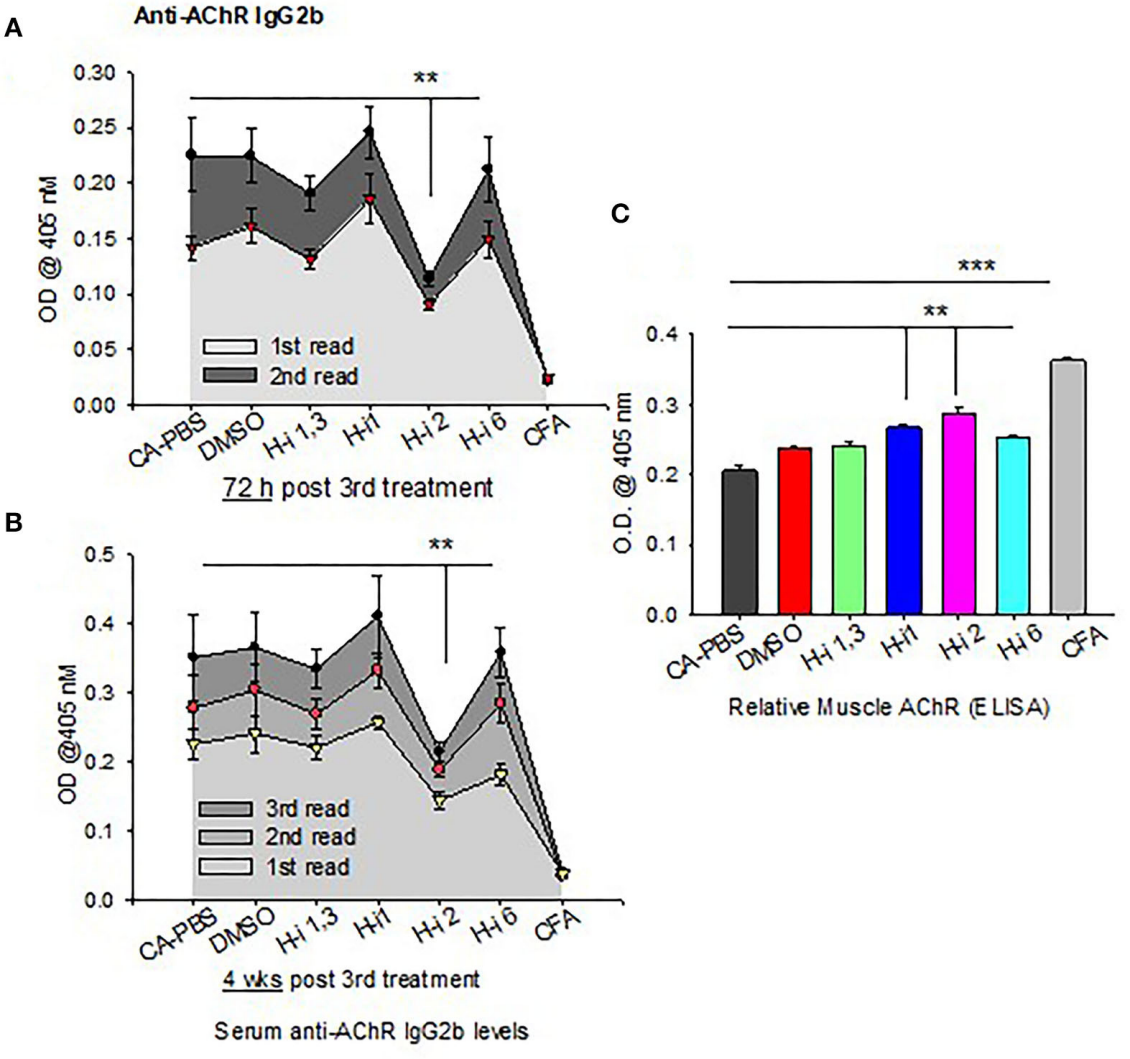
AChR-specific IgG2b levels in EAMG mice ± HDAC-i. **(A,B)** CFA (untreated) and EAMG mice were treated with HDAC-i and their blood was collected to obtain the serum at 72 h and 4 weeks post 3rd treatment. Serum dilutions were titrated to perform the ELISA for AChR-specific IgG2b (*n* = 3 for the CFA group, *n* = 5 for each CA or EAMG group). Each plot shows multiple plate readouts at a minute or less interval using 405-nm OD. **(C)** Crude preparation of muscle receptor from forelimb muscle of CFA and EAMG mice were analyzed for relative density of functional AChR *via* ELISA (using absorbance at 405 nm). **P < 0.01, ***P < 0.001, One-way ANOVA. *n* = 3 for the CFA group, *n* = 5 for each CA-PBS, DMSO, and HDAC-i treated EAMG group. The experiment is representative of three similar experiments.

IL-6 has been shown to induce plasmablast differentiation. To determine whether HDAC-i 2 mediated depletion of intracellular IL-6 and reduced frequencies of BCMA^+^ plasma cells (Figures 5A,B) correlated with the changes in autoAb levels, we examined the pathogenic anti-AChR Ab in serum from mice bled at different post treatment time points. Of the different isotypes (IgM, IgG, IgG1, and IgG2b), the complement-fixing anti-AChR IgG2b isotype in EAMG mice is the pathogenic isotype that predominantly binds to AChR at the neuromuscular junction. We found a significant reduction (40% decrease) in the anti-AChR IgG2b level of the HDAC-i 2 treated mice both 72 h and 4 weeks post 3rd treatment (Figures 6A,B). Of the various HDAC-i tested in this study, only HDAC-i 2- was effective in significantly reducing serum anti-AChR IgG2b. Unexpectedly, despite reducing sIL-6 levels significantly, HDAC-i 1 treatment slightly elevated the autoAb levels.

### Clinical Grades and Functional Muscle AChR

All mice had disease grades between 2 and 3, and they had comparable muscle strength averages at the start of the treatment. Clinical grades (as determined by blinded analyses) revealed that untreated CA mice (*n* = 5) had no change in disease characteristics or grades. All treatment groups (*n* = 5) were phenotypically alike, with milder disease grades between 1 and 2. The HDAC-i 1 treated mice had slightly higher body mass, whereas HDAC-i 2 treated mice had increased mobility than the other groups. Quantitation of AChR from proximal forelimb muscle indicates preservation of relatively high levels of functional AChR in the EAMG group treated with HDAC-i 2 (Figure 6C). Untreated CA mice had the lowest levels of muscle AChR compared to the other groups.

### nCounter Autoimmune Profiling Panel

**Figure 7:**
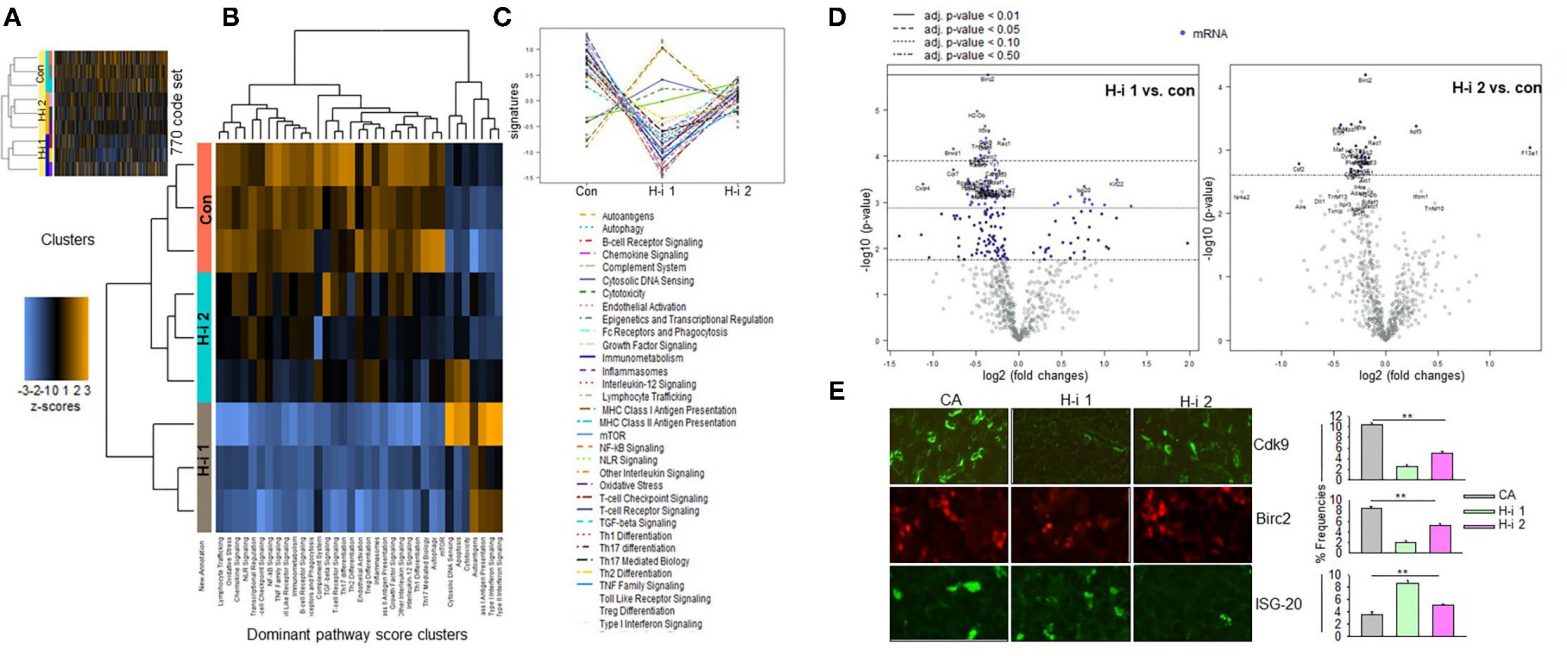
nCounter gene expression panel. Expression profiling of autoimmune-associated genes using the NanoString nCounter system. **(A)** Heatmap shows expression intensities of 770 autoimmune-associated genes clustered in rows (blue reflecting lower and brown showing higher abundance mRNAs). The columns represent pooled DMSO controls (lanes 1–3) and H2- (lanes 4–6) and H1- (lanes 7–9) treated spleen mRNA samples from EAMG mice (*n* = 5 per group, samples pooled) 4 weeks post 3rd treatment with HDAC-i. **(B,C)** *Pathway scores*. Pathway heatmap score and score plot reflect an overview of the dominant pathways affected by treatments of HDAC1 (H1) or HDAC2 (H2) relative to the baseline solvent controls (Con). Pathway average scores (shown by lines) represent the gene expression profile of each sample compressed into a small set of pathway scores. These scores are placed using the first principal component of each gene set data; in general, an increased score corresponds to increased expression. **(D)** Volcano plot depicts the distribution of the estimated log fold change or differential expression of each gene relative to the baseline or controls. Highly statistically significant genes are above the horizontal lines (*p*-value thresholds), and differentially manifested highly-repressed or highly-expressed genes are on the left or right sides, respectively. Up to 20 of the most statistically significant genes are represented by colored dots in the plot. Highly up/downregulated genes in the volcano plot are described in Tables 1, 2. **(E)** Shown are the fluorescence microscope captured images of spleen sections from DMSO, HDAC-i 1, and HDAC-i 2 treated EAMG mice. The images are representative views from the sections stained with Cdk9, IAP-1, and ISG-20 specific Alexa-conjugated Abs. A semi-quantitative assessment of fluorescence-deposits indicates relative protein abundance in samples (bar graphs). Result is representative of three independent experiments. Vertical bars represent standard error, *n* = 3 per group. **P < 0.01.

To compare the gene signatures modulated upon HDAC1 and HDAC2 inhibition, mRNA profiling analysis was performed using the NanoString nCounter Autoimmune Profiling Panel platform (NanoString Technologies). We analyzed the direct copy numbers of the mRNAs using probes for 770 genes linked to autoimmune regulation. The genes above the threshold expression counts of 20 and 50 were analyzed for differential expression or fold expression changes in the advanced mode of the software nSolver 4.0. Comparison of the spleen mRNA profiles following HDAC-i 1 vs. HDAC-i 2 treatments showed substantial heterogeneity in the expressed genes between these groups but marked homogeneity across the samples within each group in the heatmap and on the pathway score plots (Figures 7A–C). The volcano plots displaying the –log_10_ (*p*-value) and log_2_ fold change of each gene evidently showed the comparative gene expression patterns in the spleen following HDAC1 or HDAC2 inhibition (Figure 7D). We noted that the gene sets associated with specific cell signaling pathways included the B cell receptor, chemokines, Fc receptor, growth factor, inflammasome, and NF-κB, which had higher negative pathway scores (repressed) for HDAC1 inhibition, compared to lower negative scores (less repressed) for HDAC2 inhibition (Figures 7B,C, and Table 1). Additionally, the Kyoto Encyclopedia of Genes and Genomes pathway analysis, in which genes within the panel were mapped to the pathway and differential expression information was overlaid on the protein based on the pathway image, revealed significant differential expression of cytokine receptors relative to the baseline (Table 2). A qualitative immunofluorescence imaging of spleen sections was performed to confirm the altered expression of a few genes reflected in nCounter analyses. Consistent with the mRNA profiling analysis data, the images showed fewer cells stained positive for Birc2 and CDK9 in HDAC-i 1/2 and higher frequency of cells positive for ISG20 in HDAC-i 1 treated over CA control splenocytes (Figure 7E).

**Table 1:** Significantly up-/down regulated selective genes relative to baseline in the Volcano plot of (H-i)-treated spleen 4 wks post 3^rd^ treatment, **+**, more prominently down- or up regulated; -, less prominent.

**Downregulated (↓) genes**	**Function**	**H-i 1**	**H-i 2**
**Birc 2 (IAP-2**) (NM_007465.2)	Inhibitor of apoptosis	+	-
H2-Ob (NM_010389.3)	Histocompatibility 2, located in MHC Class II region	+	-
IL6ra (NM_010559.2)	Subunit of IL6R, binds to IL6 with low affinity	+	-
Rac 1 (NM_009007.2)	Pleiotropic function, upregulate glycolysis and Pentose pathways	+	+
**Cdk9** (NM_130860.3)	Critical for Polymerase II transcription initiation, elongation/termination	+	-
Nfatc1 (NM_016791.4)	Controls transcription of genes that directs cytotoxic effector function of CD8+ T cells	+	-
Brwd 1 (NM_001103179.1)	Transcriptional activator	+	-
Plcg 1 (NM_021280.3)	Phospholipase C, Gamma, overexpressed in cancer, role in cytoskeleton, cell migration	-	+
Csf2 (NM_009969.4)	Controls production, differentiation, and function of granulocytes	-	+
Dyn II (NM_026556.4)	GTP binding protein, involved with cell motility	-	+
**Upregulated (↑) genes**			
ISG20 (NM_020583.5)	Interferon-stimulated gene 20, single-stranded RNA exonuclease	+	-
Kif 22 (NM_145588.1)	Inhibitor of cell proliferation	+	-
F13a 1 (NM_001166391.1)	Encodes for a coagulation factor	-	+
IkZf3 (NM_011771.1)	Controls differentiation of lymphoid progenitor cells in to pre T and pre B cells	-	+

**Table 2:**
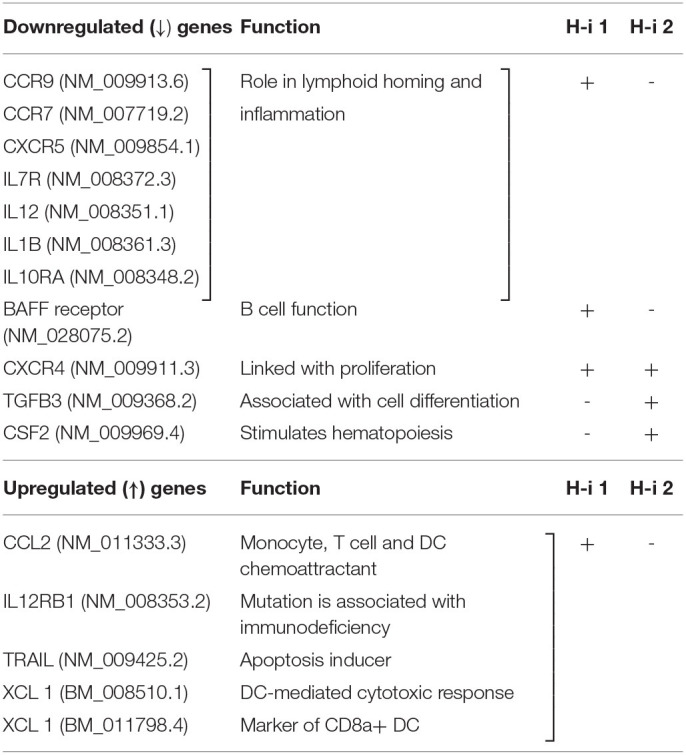
Cytokine-Cytokine receptor interaction: significantly up-/down regulated relative to baseline, +, more prominently down- or up regulated; -, less prominent.

## Discussion

HDAC isoforms are either specific genes or products of alternative splicing of a pre-mRNA transcript or single nucleotide polymorphism alloforms of an HDAC. HDAC-i are structurally diverse compounds that target one or more of these isoforms. TSA inhibits the “classical” HDACs that include class I (HDAC 1, 2, 3, and 8) and class II (HDAC 4–7, 9, and 10) isoforms (34). In this study, we sought to evaluate the effects of inhibiting HDAC class I (HDACs, 1/3, 1, and 2) and class II on the expression of key inflammatory cytokines IL-6 and IL-21 and on the production of autoAb in EAMG mice. Monoclonal antibodies (mAbs) targeting IL6 or IL6 receptor has demonstrated clinical efficacy against many diseases, including preclinical models of MG, and a new anti-IL-6 receptor (Satralizumab) is currently undergoing a clinical trial for the treatment of MG. Although mAbs produce long-term target-specific effects, stand-alone mAb therapy has some limitations, including adverse effects and possible non-sensitivity to therapy, e.g., unaltered autoantibody levels or clinical pathology following B-cell specific therapy in many patients with MG (35). Therefore, it might be beneficial to explore through research the potential of alternative treatments targeting the same molecules, in particular therapies that utilize natural molecules like many HDACi.

The present study represents the first step toward identifying the role of HDAC isoforms in abnormal inflammation associated with MG pathogenesis in an EAMG mouse model. HDACs, often triggered and activated by specific environmental stimuli, have been previously implicated in autoimmunity (36, 37). Recent studies have shown that HDAC-i such as TSA, vorinostat, and valproic acid reduce the disease severity of many autoimmune diseases (25, 27, 30). Consistent with the findings of these studies, we show here that the dysregulated inflammatory response in EAMG, and also in MG, is due at least in part to epigenetic changes involving histone acetylation modifications. In our analysis, significant increase in HDAC expression in EAMG mice compared to CFA controls evidently implies a potential role of HDACs in MG pathogenesis. Furthermore, HDAC-inhibition-mediated decreases in AChR-induced sIL-6 levels in concert with IL-6 mRNA suggest that HDACs regulate the secretory levels of IL-6 at the transcriptional level.

According to published studies, HDAC inhibition reduces the expression of the inflammatory cytokine IL-6 (38). We also found that inhibiting certain HDACs reduced IL-6 (mRNA and protein) as well as circulating autoAb levels. In our study, the isoform-selective function of HDACs in regulating IL-6 was exhibited even within the same class of HDACs. We demonstrated that inhibition of HDAC1, but not HDAC2, is most effective in reducing the inducible levels of secretory IL-6. Conversely, as shown in the flow cytometry data, inhibition of HDAC2, but not HDAC1, completely exhausts intracellular IL-6 in PBMCs. HDAC2 inhibition appears to deplete intracellular IL-6 by a mechanism distinct from other HDAC-i, likely through one of the following:(a) prompt release of intracellular IL-6 followed by transcriptional inactivation through the repression of histone hyperacetylation involving H4K20ac, (b) stabilizing IL-6 mRNA, or (c) activating intracellular degradation of IL6 by the proteasomal pathway. Of note, there were slight differences in IL-6 production from stimulated PBMCs or splenocytes from HDAC-i-treated and untreated mice terminally bled 4 weeks post 3rd treatment. The result indicates that the inhibitory effect of HDAC-i on IL-6 wanes over time in the absence of further treatment.

IL-6 has long been demonstrated to be a potent activator of B cells and an inducer of plasma cell differentiation (39). IL-6 has also been reported to induce IL-21 in CD4 cells, which, in turn, triggers follicular Th cell and plasmablast differentiation; thus, IL-21 has known to play an important role in IL-6-mediated Ab production from B cells. However, in our experiments, HDAC-i reduced levels of IL-6, but not IL-21, in the supernatant of AChR-stimulated PBMCs from HDAC-i treated EAMG mice, suggesting that IL-21 expression is possibly not regulated by HDACs. Interestingly, reduction in the intracellular IL-6 following inhibition of HDAC2 was also associated with a decrease in the levels of autoAb and corresponding decrease in the frequency of BCMA^+^ plasma cells, suggesting a role for IL-6, independent of IL-21 in B cell activation. Thus, intracellular IL-6 rather than sIL-6 appeared to be more important in inducing autoAb production. Notably, despite reducing the sIL-6 the most, HDAC-i 1 had no effect on autoAb levels. Although without continued treatment with HDAC-i, levels of both sIL-6 and intracellular IL-6 from stimulated PBMCs or splenocytes tend to increase over time, the autoAb-reducing effect of HDAC-i 2 treated mice persisted until the experimental endpoint (4 weeks post 3rd treatment).

Muscle fatigue in MG occurs mainly due to autoAb-mediated loss of functional AChRs in the postsynaptic neuromuscular junctions. Anti-AChR autoAbs in MG are commonly the binding Abs that activate complements upon binding with AChRs, form membrane attack complex (MAC) and deplete AChR from the muscle endplate. EAMG-associated cytokines, including IL-6, also promote the activation of complement factors (3). Modulating autoAbs cross-link AChRs to cause its internalization and degradation, resulting in reduced surface expression of functional AChR. Blocking Abs (less common) bind to AChRs to prevent binding of ACh with the receptors (40). The complement-fixing IgG2b anti-AChR isotype is predominantly involved in EAMG pathogenesis. We found that reduced IgG2b levels in mice by HDACi-2 treatment were also associated with increased muscle AChRs and MG disease improvement.

The exact mechanism by which HDAC2 inhibition reduces anti-AChR IgG2b levels is unclear. A previous study demonstrating HDAC-i (ITF2357) mediated reduced IL-6 receptor expression on naïve CD4^+^T cells that leads to polarization of Th17 cells to Treg cells in experimental colitis (18) is a likely mechanism of autoAb reduction. In addition, by inhibiting plasmablast proliferation, HDAC-i 2 may decrease the Ab-secreting plasma cell population as indicated by higher BCMA^+^ B cells in our experiments. We found that HDAC inhibition increased H3K4 acetylations, implying that HDAC inhibition induces the transcriptional activation of some genes, while simultaneously suppressing some other genes, likely through acetylating different lysine residues of histones, such as H4K20 in our experiment.

Finally, to gain insight into the genes sensitive to HDAC1 and HDAC2 inhibition, we compared the mRNA abundance profiles in the spleen of EAMG mice 4 weeks post treatment. The nCounter mRNA analysis revealed genes modulated by HDAC1 or HDAC2 inhibition. Despite limited fold changes in gene expression, heatmap and scatter plot distinctly showed differentially and similarly expressed several genes and intensity of gene expression following HDAC-i treatment. Genes associated with inflammation, transcription, or induction of dendritic cell antigen processing (e.g., IL-6R alpha, Cdk9, and H2-ob, respectively) were downregulated in HDAC-i 1 treated samples. Both HDAC1 and HDAC2 inhibition, however, downregulated the expression of other genes, such as Birc2 (apoptosis inhibitor), Brwd1 (transcriptional activator), Rac 1 (pleiotropic function), and inflammatory cytokines and cytokine receptor genes (pathway score analysis). The pathway plot also revealed that HDAC1 inhibition is associated with the upregulation of certain pathways (e.g., antigen presentation, type 1 and type II interferon) that counteract autoimmune improvement. In contrast, HDAC2 inhibition moderately suppressed the inflammatory pathways and scored lesser in inducing the pathways associated with autoimmunity. Thus, the pathway score plots further explain the differential anti-AChR autoAb response outcomes induced by HDAC1 and HDAC2 inhibition. Further studies on the role of HDACs and other epigenetic readers, such as bromodomains, may help identify specific epigenetically regulated targets for MG therapy.

## Conclusion

In summary, the present study demonstrated both functionally distinct and overlapping biological functions of HDAC isoforms pertinent to inflammatory and autoantibody signaling pathways in a mouse model of MG. Targeting specific HDAC isoform(s), ideally, direct targeting of gene(s) in the HDAC downstream pathway, either independently or in combination with existing MG therapies, may prove to be beneficial. Future investigations are needed to evaluate these molecules as new anti-inflammatory drug targets to potentially improve MG treatment efficacy and clinical application.

## Data Availability Statement

The original contributions presented in the study are included in the article/Supplementary Material, further inquiries can be directed to the corresponding author.

## Ethics Statement

The studies involving human participants were reviewed and approved by Institutional Review Board of the UTMB. The patients/participants provided their written informed consent to participate in this study. The animal study was reviewed and approved by UTMB Animal Care and Use Committee.

## Author Contributions

RH: conceptualization and methodology, supervision, project administration, data curation, formal analysis, validation, and writing-original draft. AB, NI, MI, ZW, MJ, and RH: investigation. AB, MI, MJ, LY, LS, and XF: writing-review and editing. RH and XF: funding acquisition. All authors have read and approved the final version of the manuscript for publication.

## Funding

This work was primarily supported by a Pilot Research Award from Conquer Myasthenia Gravis (the United States) to RH and XF. RH was supported in part by AFM-Telethon. XF was supported by an endowment fund from UTMB.

## Conflict of Interest

The authors declare that the research was conducted in the absence of any commercial or financial relationships that could be construed as a potential conflict of interest.

## Publisher's Note

All claims expressed in this article are solely those of the authors and do not necessarily represent those of their affiliated organizations, or those of the publisher, the editors and the reviewers. Any product that may be evaluated in this article, or claim that may be made by its manufacturer, is not guaranteed or endorsed by the publisher.

## Abbreviations

AChR: Acetylcholine receptor
AutoAb: Autoantibody
BCMA: B cell maturation antigen
EAMG: Experimental autoimmune myasthenia gravis
HAT: Histone acetyltransferase
HDAC: Histone deacetylase
HDAC-i 1: Inhibitor targeting histone deacetylase 1
HDAC-i 2: Inhibitor targeting histone deacetylase 2
HDAC-i 6: Inhibitor targeting histone deacetylase 6
HDAC-i: Histone deacetylase inhibitor
MG: Myasthenia gravis
sIL-6: Soluble IL-6.
