# Supplementary figures and images for: Histone Deacetylase Isoforms Differentially Modulate Inflammatory and Autoantibody Responses in a Mouse Model of Myasthenia Gravis
Source: Front Neurol. 2022 Feb 10;12:804113. doi: 10.3389/fneur.2021.804113 (PMC8866970; doi:10.3389/fneur.2021.804113)

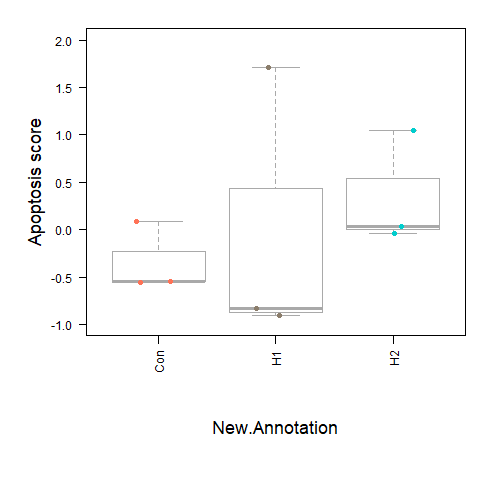

Supplement: Supplementary file 1 [file Presentation_1.zip › Apoptosis pathway scores vs. New.Annotation.png]

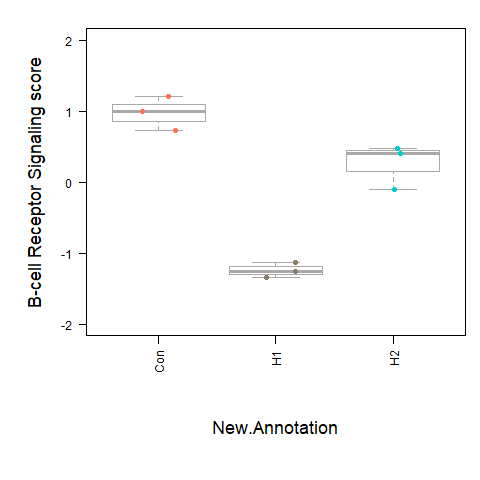

Supplement: Supplementary file 1 [file Presentation_1.zip › B-cell Receptor Signaling pathway scores vs. New.Annotation.png]

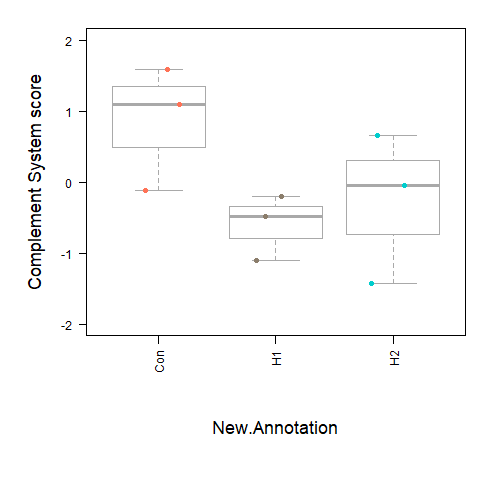

Supplement: Supplementary file 1 [file Presentation_1.zip › Complement System pathway scores vs. New.Annotation.png]

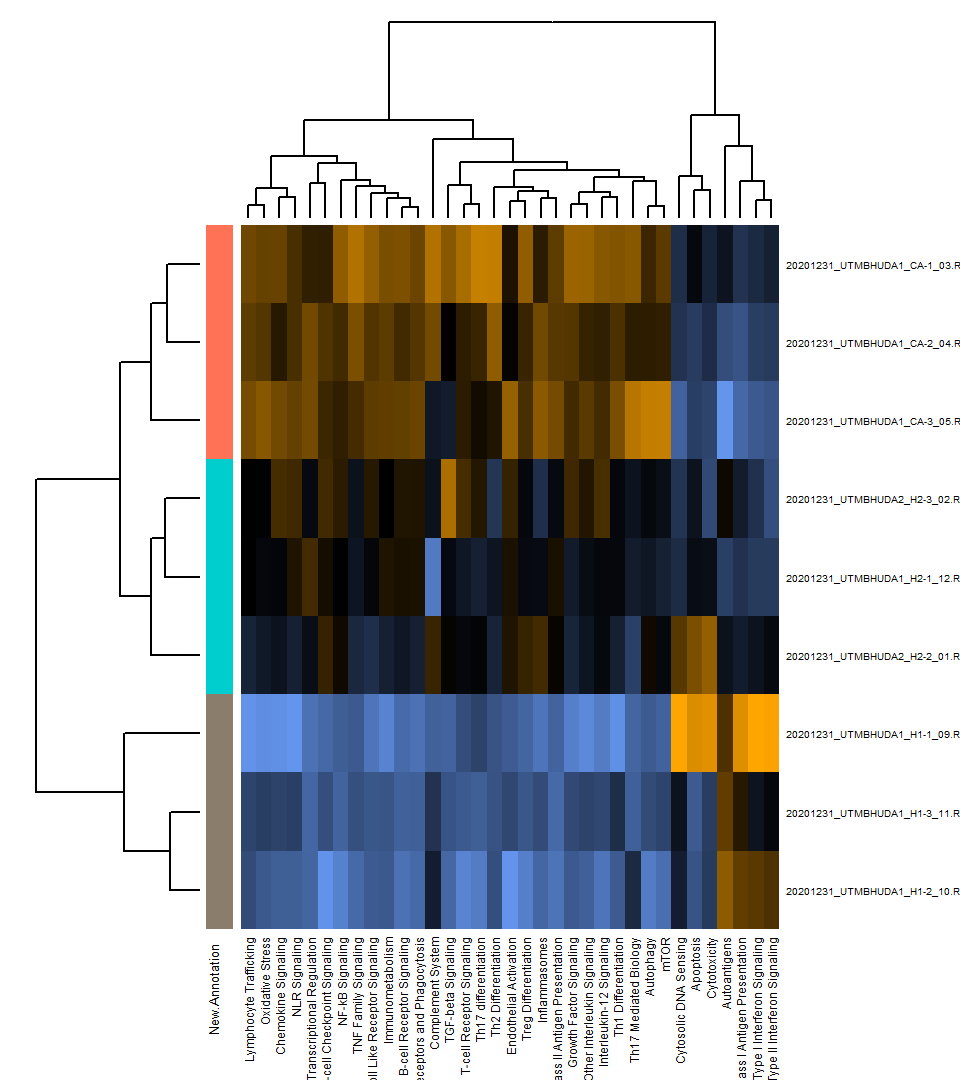

Supplement: Supplementary file 1 [file Presentation_1.zip › heatmap of signatures.png]

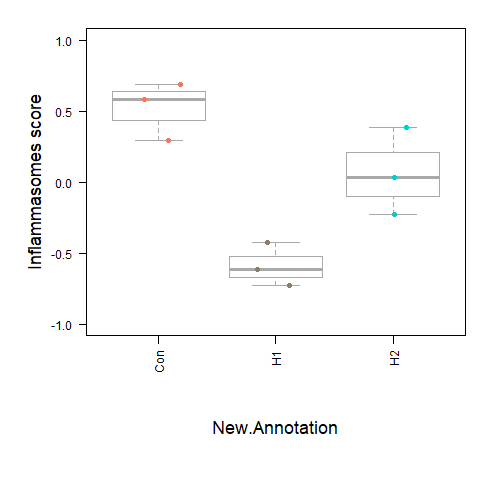

Supplement: Supplementary file 1 [file Presentation_1.zip › Inflammasomes pathway scores vs. New.Annotation.png]

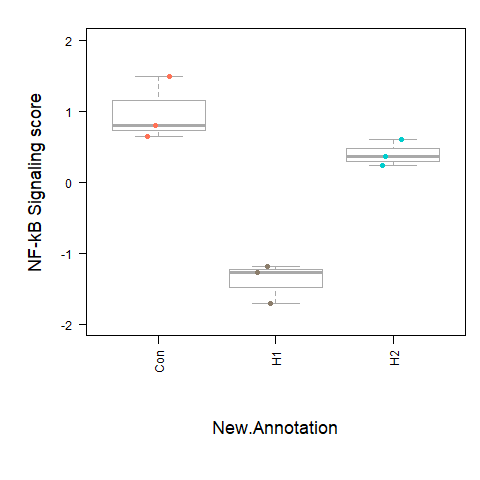

Supplement: Supplementary file 1 [file Presentation_1.zip › NF-kB Signaling pathway scores vs. New.Annotation.png]

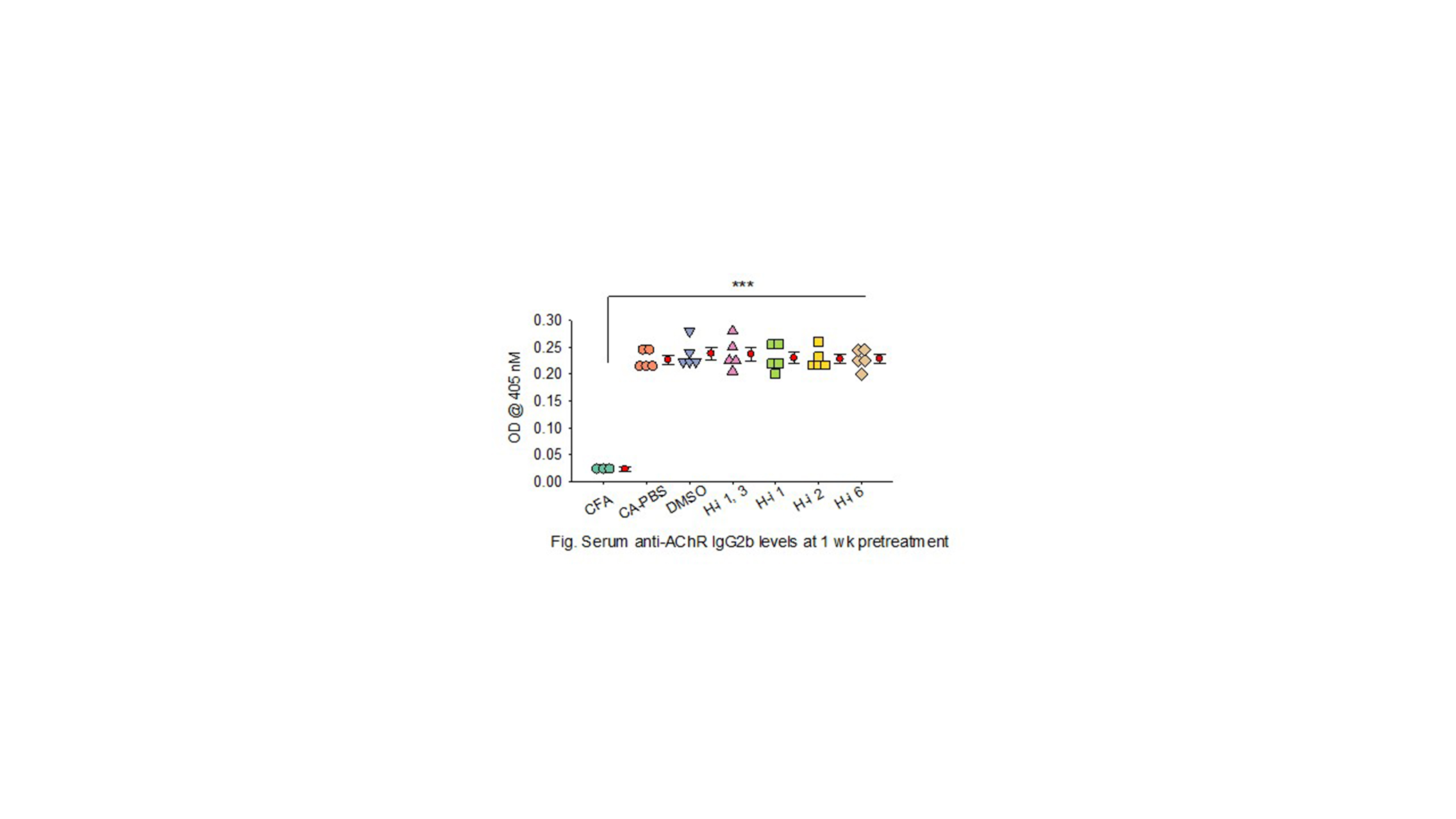

Supplement: Supplementary file 2 [file Image_1.jpg]
